# The bidirectional interplay between ncRNAs and methylation modifications in gastrointestinal tumors

**DOI:** 10.7150/ijbs.87028

**Published:** 2023-09-11

**Authors:** Minyu Kong, Xiao Yu, Wenzhi Guo, Ran Guo

**Affiliations:** 1 Department of Hepatobiliary and Pancreatic Surgery, The First Affiliated Hospital of Zhengzhou University, Zhengzhou, China.; 2 Henan Liver Transplantation Centre, China.; 3 Henan Organ Transplantation Quality Control Centre, China.; 4 Open and Key Laboratory for Hepatobiliary & Pancreatic Surgery and Digestive Organ Transplantation at Henan Universities, China.; 5 Henan Innovative Research Group for Hepatobiliary & Pancreatic Surgery and Digestive Organ Transplantation, China.

**Keywords:** ncRNAs, methylation, GI tumors, Mechanism, Biomarker and Therapy

## Abstract

The aberrant expression of methylation and ncRNAs, two crucial regulators of epigenetic modifications, has been widely demonstrated in cancer. The complex interplay between them is essential in promoting malignant phenotype, poor prognosis, and drug resistance in GI tumors (including esophageal, gastric, colorectal, liver, and pancreatic cancers). Therefore, we summarize the interrelation process between ncRNAs and methylation modifications in GI tumors, including the detailed mechanism of methylation enzyme regulation of ncRNAs, the molecular mechanism of ncRNAs regulation of methylation modifications, and the correlation between the interactions between ncRNAs and methylation modifications and clinical features of tumors. Finally, we discuss the potential value of ncRNAs and methylation modifications in clinical diagnosis and therapy.

## 1. Introduction

Cancer remains a serious global public health issue, with digestive system cancers accounting for a large portion of all cancers. According to statistics, it is estimated that there will be 4.82 million new cancer cases in China in 2022, with digestive system cancers accounting for as much as 42.5% 1. In 2021, digestive system cancers were also the most common cancer type in the United States, accounting for 25% of all new cancer cases 2. Digestive system cancers include esophageal cancer, gastric cancer, colorectal cancer, liver cancer, pancreatic cancer, and gallbladder cancer. In addition to their high incidence, the prognosis for digestive system tumors is often poor 3. According to global cancer statistics in 2020, the 5-year survival rate for pancreatic cancer is less than 10%, while the 5-year survival rate for esophageal and liver cancer is less than 20%, indicating an abysmal prognosis 4. The high incidence and mortality of digestive system tumors are mainly due to the lack of currently available effective diagnostic and treatment methods 5. For example, there is a lack of widely available screening methods for esophageal cancer, and patients are often diagnosed with cancer in the late stages 6. Therefore, understanding the pathogenesis of cancer, identifying biomarkers that aid in the early diagnosis of digestive tract tumors, and finding therapeutic targets are of great significance in reducing the incidence of digestive tract tumors and improving prognosis.

NcRNAs are a class of RNA molecules that do not encode proteins, and they play an important role in regulating cellular transcription and post-transcriptional processes 7. Based on their length, structure, and function, ncRNAs can be divided into several types, including miRNA, lncRNA, circRNA, and piRNA 8, 9. NcRNAs play a significant role in regulating gene expression in digestive system tumors. They use their complex epigenetic regulatory mechanisms to act as upstream regulators of downstream oncogenes or tumor suppressor genes 10. For example, in liver cancer, circMDK upregulates ATG16L1 by inhibiting miR-346 and miR-874-3p, which promotes the occurrence and development of liver cancer 11. LncRNA AGPG has been shown to play a role in esophageal cancer by binding to and stabilizing PFKB3, which increases glycolytic flux and cell cycle progression in esophageal cancer cells. 12. Moreover, ncRNAs are also subject to epigenetic regulation and play a role in cancer. For instance, in colorectal cancer, m6A methyltransferase catalyzes the maturation of miR-124, which promotes colorectal cancer metastasis through the miR-124/SPRED2/MAPK axis 13. These studies highlight the crucial role of ncRNAs in regulating digestive system tumors, as they can act as intermediate molecules to link many cancer-related molecules together.

In recent years, there have been increasing studies on epigenetic modifications 14. Epigenetic modifications refer to a genetic regulatory mechanism that affects gene expression without changing DNA sequence 15. In cancer, epigenetic modifications can affect gene expression and function by modifying DNA, histones, RNA, ncRNAs, etc., thereby affecting tumor development 16. There are many epigenetic modifications currently known, including methylation 17, acetylation 18, ubiquitination 19, and others. Among them, methylation is the most studied, as it is a common modification in cell activity and can change the expression level or structural function of DNA, RNA, and proteins 20. Many studies have found that methyltransferases can regulate tumor progression and drug resistance in the digestive system by catalyzing methylation or demethylation of cancer-related genes 21.

**Figure 1:**
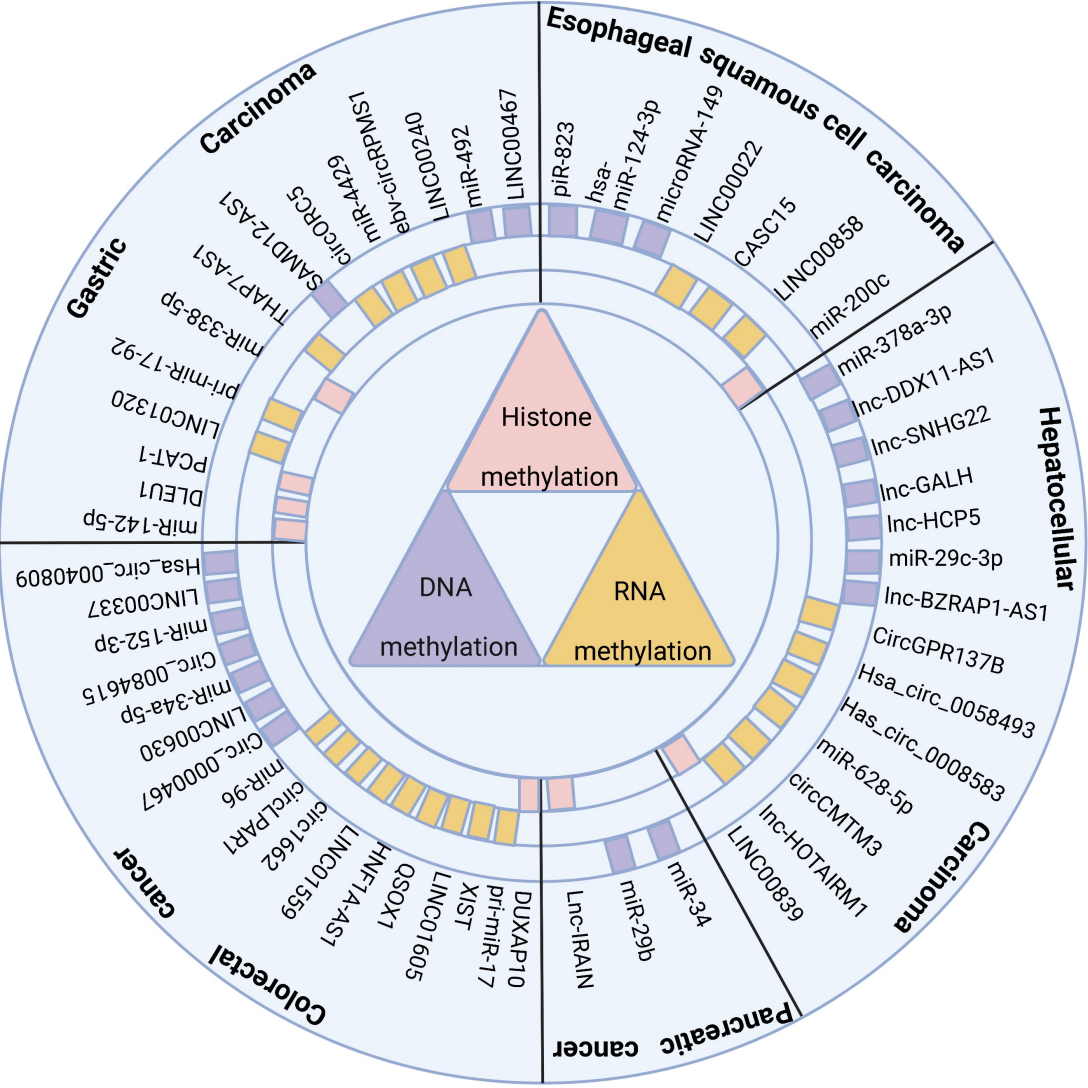
ncRNAs regulate cancer progression through DNA methylation, RNA methylation, and histone methylation in digestive system tumors

NcRNAs and methylation modifications are crucial factors that affect the occurrence and development of digestive system cancer, and exploring their interaction is valuable. Thus, this paper summarizes the common methylases and their interacting ncRNAs in recent years in digestive system tumors (Figure 1), examines the mechanism of interaction between them, and their impact on tumor occurrence and development, including the clinical features of tumors and their biological processes. Hopefully, this review will provide novel insights for studying ncRNAs associated with methylation modification and identify new clinical diagnostic markers and therapeutic targets.

## 2. Methyltransferases

Methylation is an essential modification of nucleic acids and proteins. Specific sites on nucleic acids and proteins are methylated by the catalysis of methyltransferases, resulting in changes in gene expression or protein structure and function 22. In this section, we will introduce several methylesterases commonly found in digestive tumors, which are classified into three categories according to the methylation modification substrate: DNA, histone, and RNA methyltransferases.

### 2.1 DNA methyltransferases

DNA methylation is adding a methyl group to the fifth carbon atom of cytosine on DNA, producing 5-methylcytosine (5-mC) 23. The DNA methyltransferases (DNMTs) involved in the association between digestive system tumors and ncRNAs include DNMT1, DNMT3A, and DNMT3B. DNMT1 is the most abundant DNA methyltransferase, responsible for maintaining the existing DNA methylation pattern. It is often overexpressed in digestive system tumors, resulting in a highly methylated global or local state 24, 25. On the other hand, DNMT3A and DNMT3B are often mutated or deleted in tumor cells, leading to a globally or locally hypomethylated state 26.

### 2.2 Histone methyltransferases

Histone methylation usually refers to the addition or removal of methyl groups on lysine residues of histones, which affects the structure and function of chromatin 27. Common histone methyltransferases associated with ncRNAs in digestive system tumors include EZH2, RIZ1, and LSD1. EZH2 is often overexpressed or mutated in cancer, and it silences some tumor suppressor genes by methylating H3K27 to H3K27me3 28. H3K27 is a lysine residue and the 27th amino acid of histone H3 29. RIZ1 can methylate H4R3 to H4R3me1/2; its low expression or loss in cancer is a principal element in cancer cell biology 30. LSD1 is slightly different from the first two methyltransferases. A histone demethylase can remove methyl groups from H3K4me1/2 or H3K9me1/2. It is often overexpressed in cancer and is associated with poor prognosis in multiple cancers 31-33.

### 2.3 RNA methyltransferases

RNA methylation mainly involves the methylation modification of bases or sugar rings on RNA molecules, which can alter the structure and function of RNA 34. The RNA methyltransferases associated with ncRNAs in digestive system tumors are METTL3, METTL14, WTAP, and FTO. METTL3 and METTL14 are m6A writers, which can transfer methyl groups to adenosine on RNA molecules. However, their expression and functions vary in tumor tissues 35. WTAP does not have a methylation function, but it is an essential auxiliary factor of m6A methyltransferase and can form a complex with METTL3 and METTL14 to regulate m6A modification 36. FTO, on the other hand, is a demethylase that can remove m6A methylation modification on RNA molecules. FTO is overexpressed in various cancers and promotes biological processes such as tumor cell proliferation, angiogenesis, and drug resistance by reducing m6A levels 37-39.

## 3. Methylation to ncRNAs

In this section, we will mainly introduce the mechanism by which methyltransferases actively regulate ncRNAs involved in the occurrence and development of digestive system tumors. We will also discuss the impact of this regulation on the biological functions of tumor cells (Table 1).

### 3.1 DNA methylation modifies ncRNAs

Research has found that many ncRNA promoters contain abundant CpG sites, which makes these ncRNAs susceptible to regulation by DNA methyltransferases 40, 41. In esophageal cancer, it has been shown that miR-149 can inhibit the growth and invasiveness of ESCC cells by suppressing the RNF2/Wnt/β-catenin pathway. However, DNMT3B methylates the miR-149 promoter and suppresses miR-149 expression, reversing its anti-cancer effect 42. Additionally, DNMT1 indirectly regulates BCAT1 by methylating miR-124-3, which directly targets BCAT1 to affect the proliferation and migration of esophageal cancer cells 43. BCAT1 is an enzyme that converts the α-amino group of branched-chain amino acids to a-KG and is highly correlated with the occurrence and development of various cancers 44. DNMT1 indirectly regulates BCAT1 by methylating miR-124-3. In liver cancer, it was found that DNMT1 methylates miR-16-5p to play a carcinogenic role. Interestingly, as a lncRNA, SNHG22 recruits DNMT1 to the promoter region of miR-16-5p through interaction with EZH2. In this axis, ncRNAs actively regulate methyltransferases while also being catalyzed by them 45. Additionally, it has been found that DNMT1 silences miR-378a-3p by methylating it, thereby promoting the expression of TRAF1 and activating the NF-κB signaling pathway. The p65 in the NF-κB signaling pathway can promote the upregulation of DNMT1, thereby forming a positive feedback loop 46. Furthermore, in colorectal cancer, miR-152-3p was found to upregulate and inhibit TMSB10, while upregulation of DNMT1 can reverse the effect of miR-152-3p on CRC tumor growth 47. DNA methyltransferases mainly regulate the occurrence and development of digestive system tumors by indirectly affecting various oncogenes by regulating miRNA promoter methylation.

### 3.2 Histone methylation modifies ncRNAs

Many studies have found that histone methylation plays a vital role in regulating ncRNA expression 48. In esophageal cancer, EZH2, as a histone methyltransferase, promotes esophageal cancer cells' invasion and EMT process by upregulating miR-200c. Although the mechanism of regulating cells by EZH2 and miR-200c is not yet precise, they are still promising biomarkers for treating esophageal cancer patients 49. In addition, in gastric cancer, embryonic ectoderm development protein (EED) can suppress the expression of miR-338-5p by promoting histone methylation. EED has been confirmed to be responsible for the methylation of histone H3K27 50. Knockdown of EED promotes miR-338-5p expression, inhibiting the proliferation and invasion of GC cells. Interestingly, miR-338-5p can inhibit the expression of METTL3 and regulate the methylation level of CDCP1. EED can promote CDCP1 expression and GC development through this axis 51. Another study found that histone methyltransferase RIZ1 can mediate the enrichment of H3K9me1 on the promoter of lncRNA HOTAIRM1, promoting the growth and metastasis of HCC cells 30. In addition to histone methyltransferases, histone demethylases also have research on regulating ncRNAs. LSD1 is an enzyme that regulates the demethylation of H3K4me1/2 and H3K9me1/2 52. Studies have found that high expression of LSD1 significantly promotes the migration and EMT process of GC cells. Mechanistically, LSD1 promotes miR-142-5p expression and downregulates CD9 through its demethylation function 53. Overall, histone methylation plays an essential role in digestive system tumors by regulating the expression of ncRNAs, and targeting various histone methyltransferases is a promising therapeutic approach.

### 3.3 RNA methylation modifies ncRNAs

RNA methylation modification occurring on ncRNAs mainly involves m6A methylation. The m6A methyltransferases METTL3 and METTL14 play an important role in regulating ncRNAs in digestive system tumors 54, 55. In gastric cancer, METTL3 was found to catalyze m6A in an m6A/DGCR8-dependent manner to process pri-miR-1792 into a mature form. MiR-1792 activates the AKT/mTOR pathway to promote gastric cancer cell resistance by targeting PTEN or TMEM127 56. LncRNA THAP7-AS1 was shown to promote GC cell progression. At the same time, THAP7-AS1 was upregulated by METTL3-mediated m6A modification, which enabled CUL4B protein entry into the nucleus by facilitating the interaction of nuclear localization signal (NLS) with input protein α1. CUL4B indirectly regulates the PI3K/Akt pathway in the nucleus by regulating miR22-3P and miR-320a. In this axis, METTL3 and the methylation regulation of ncRNAs by CLU4B play an important role in the development of gastric cancer 57. In addition, METTL3 upregulates the expression of LINC01559 and circQSOX1 by methylating them, and these two ncRNAs also promote colorectal cancer progression by sequestering miRNAs 58, 59. Additionally, METTL3 upregulates the expression of circ 1662 by binding to its flanking sequences and adding m6A modifications, while circ1662 promotes CRC invasion and migration by upregulating the YAP1 protein 60. METTL3 promotes cell proliferation and migration in liver cancer through the circ_0058493/YTHDC1 axis 61. The role of METTL14 is similar to METTL3, as studies have shown that METTL14 promotes the proliferation and invasion of CRC cells by regulating the m6A modification of lncRNA XIST. It was also found that XIST can be recognized and degraded by the m6A reader protein YTHDF2. Upregulation of XIST is achieved through METTL14-mediated RNA degradation inhibition of YTHDF2 62. Furthermore, in HCC, it was found that METTL14 upregulates the expression of circFUT8 through m6A modification. CircFUT8 promotes HCC progression through the miR-552-3p/CHMP4B axis. However, M1 macrophages can reverse this process by secreting miR-628-5p into HCC cells to target and inhibit METTL14 63. Additionally, WTAP plays an important role in m6A modification by forming a complex with methyltransferases. Research has found that WTAP regulates m6A modification of circCMTM3 in liver cancer. The silencing of CircCMTM3 inhibits PARK7 expression by binding to IGF2BP1, inducing iron death in liver cancer cells 64. FTO, as an m6A demethylase, is also important. In esophageal cancer, FTO reduces the m6A methylation level of LINC00022 and promotes its upregulation. The mechanism is that FTO slows down the decay rate of LINC00022 in a YTHDF2-dependent manner. LINC00022 directly binds to the p21 protein, promoting its degradation and inducing esophageal cancer cell proliferation and progression 65. Additionally, in liver cancer, a study found that FTO indirectly inhibits miR-4739 by demethylating circGPR137B, while miR-4739 can inhibit FTO expression, forming a positive feedback loop 66.

**Figure 2:**
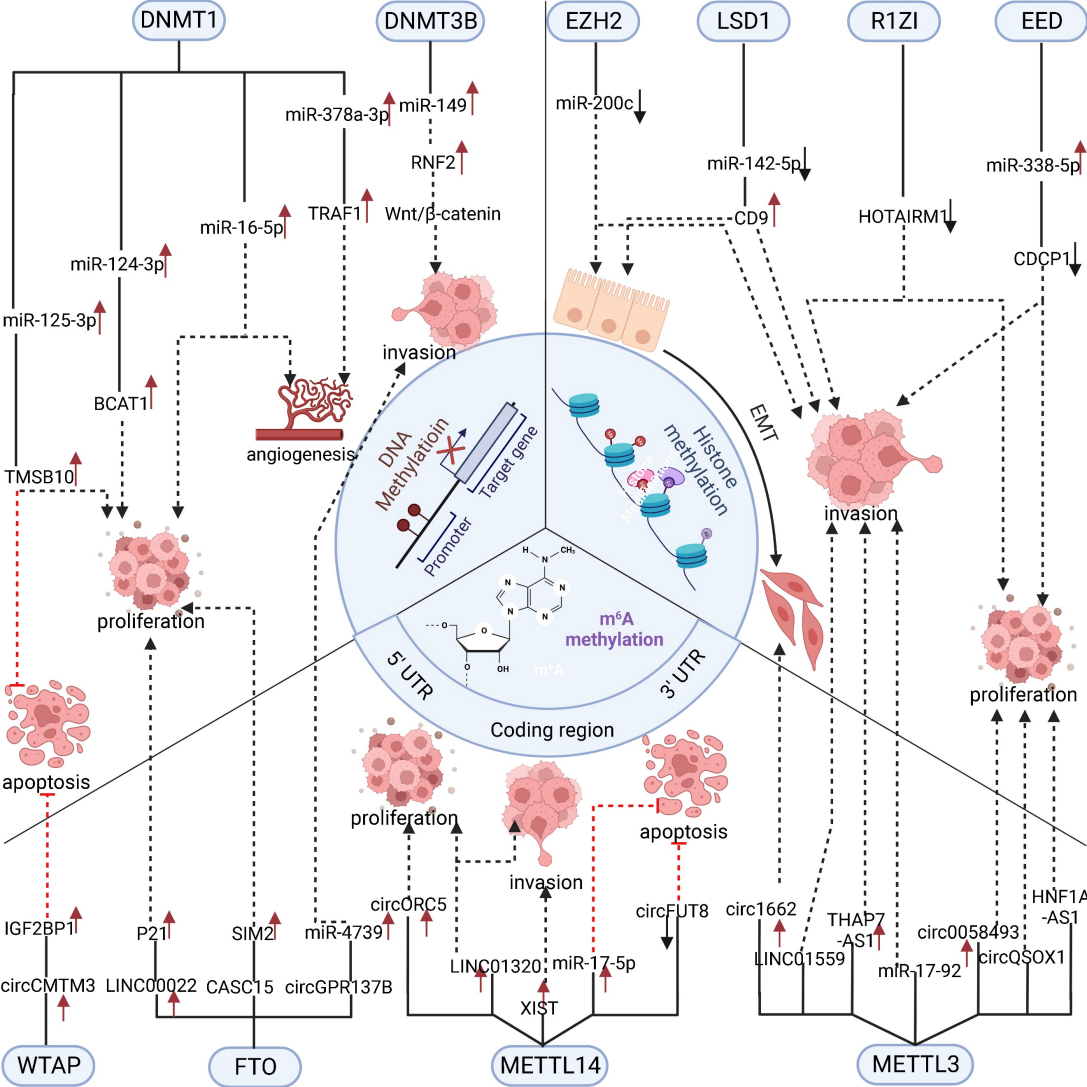
Three methylation-modified enzymes regulate the progress of related cell biology by actively regulating ncRNAs. DNA methylase DNMT1 and DNMT3B promote cancer progression through functional up-regulation of downstream microRNA. In contrast, RNA methylase mainly promotes cancer progression through the up-regulation of downstream oncogenic microRNA, lncRNA, and circRNA. Histone methylase promotes cancer progression by down-regulating the expression of related cancer suppressor microRNA.

In summary, RNA methylation modification of ncRNAs plays a vital role in regulating digestive system tumors 67. However, current research has mainly focused on m6A methylation, and it is believed that more types of RNA methylation modifications regulating ncRNAs will be discovered in the future (Figure 2).

## 4. ncRNAs to methylation

In this section, we will discuss the mechanism by which ncRNAs actively regulate methylation modification and their impact on the biological processes of cancer cells. We will divide ncRNAs into miRNA, lncRNA, and circRNA and describe their roles in regulating methylation in digestive system tumors (Table 2).

### 4.1 miRNA regulates methylation modification

MiRNAs are a class of non-coding RNAs approximately 21-23 nucleotides long, which regulate gene expression by binding specifically to the target messenger RNA (mRNA) and inhibiting its transcription 68. In digestive system tumors, miRNAs mainly regulate cancer progression by inhibiting methyltransferases. In gastric cancer, miR-492 promotes gastric cancer cells' stemness and invasion ability by directly inhibiting DNMT3B 69. In addition, miR-4429 directly targets and inhibits METTL3, regulating gastric cancer progression. METTL3 can stabilize SEC62 in liver cancer cells by m6a modification to promote cell proliferation 70. Research has shown that miR-338-5p inhibits METTL3 expression to suppress the proliferation and migration of GC cells. However, miR-338-5p is inhibited by LINC00240 71. In colorectal cancer, miR-515-5p directly inhibits DNMT1, while circ_0040809 competes with miR-515-5p to restore DNMT1 expression 72. MiR-34a-5p can be transported to CRC cells through extracellular vesicles derived from mesenchymal stem cells (MSC-EVs) and directly inhibits c-MYC to regulate DNMT3a expression. DNMT3a can regulate CRC progression by promoting PTEN methylation 73. In addition, miR-96 promotes FTO upregulation by downregulating AMPKα2 in CRC, and FTO inhibits MYC expression through demethylation modification. The inhibition of miR-96 in CRC cells' malignant phenotype is achieved by regulating FTO demethylation modification 74. In HCC, miR-29c-3p directly inhibits DNMT3B, while DNMT3B regulates HCC progression by methylating LATS1. LATS1 has been identified as a tumor suppressor gene, and its inhibition by DNMT3B promotes HCC cell proliferation and migration 75, 76. Furthermore, LINC00839 upregulates WTAP to promote liver cancer cell proliferation and invasion through the sponge-like effect of miR-144-3p. MiR-144-3p can directly inhibit WTAP from exerting its effect 77. In pancreatic cancer, miR-29b targets and inhibits DNMT3B to suppress pancreatic cancer cell apoptosis 78. In summary, in digestive system tumors, miRNAs play a role by directly targeting the mRNA of methyltransferases. Additionally, miRNAs often act as tumor suppressors to regulate the pro-cancer effect of methyltransferases.

### 4.2 lncRNA regulates methylation modification

LncRNAs are ncRNAs with a length greater than 200 bp, and their mechanism of action is complex 79. Compared to miRNAs that directly bind to methyltransferase mRNA to regulate its expression level, lncRNAs can affect the occurrence and development of digestive system tumors by regulating the expression or activity of methyltransferase in multiple ways. For example, lncRNA HCP5 indirectly promotes the expression of DNMT3A by sponging miR-29b-3p in HCC cells, and DNMT3A promotes the migration and invasion ability of HCC cells by methylating and activating the AKT pathway 80. In addition to indirectly regulating the expression level of methylation enzymes, lncRNAs can also regulate methylation enzymes in other ways. In ESCC, LINC00858 upregulates FTO expression by recruiting ZNF184. FTO promotes the proliferation, invasion, and migration characteristics of ESCC cells by demethylating MYC and upregulating its expression 81. Besides indirectly regulating the expression of methylesterases, lncRNAs can also act by recruiting methylesterases to the binding sites of target genes, and lncRNAs can directly catalyze epigenetic modifications of methylesterases to alter their activities. For example, in gastric cancer, lncRNAs SAMD12-AS1 and LINC00467 can directly interact with DNMT1 to promote DNMT1-catalyzed p53 and Reprimo promoter methylation, respectively, to regulate gastric carcinogenesis and development 82, 83. Furthermore, lncRNA DLEU1 can promote gastric cancer cell proliferation by upregulating KLF2 by recruiting LSD1 to the KLF2 promoter region 84. In colorectal cancer, the knockdown of LINC00337 inhibited angiogenesis and proliferation of CRC cells because LINC00337 inhibited CNN1 expression by recruiting DNMT1. In contrast, CNN1, a critical oncogenic factor in colorectal cancer, was upregulated by the knockdown of LINC003377 85, 86. Comparatively, LINC01605 can promote m6A modification of SPTBN2 by binding to METTL3, and LINC01605 itself is regulated by SMYD2-EP300-mediated histone methylation modifications 87. In hepatocellular carcinoma, lncRNA BZRAP1-AS1 promotes the malignant phenotype of HCC cells by interacting with DNMT3b to downregulate THBS1^88^. According to another study, lncRNA DDX11-AS1 promoted LATS2 methylation by interacting with EZH2 and DNMT1. Knockdown of DDX11-AS1 promoted LATS2 expression and thus inhibited the proliferation and invasion of HCC cells 89. Moreover, besides the recruitment of methylesterase, it was found that lncRNA DUXAP10 could also inhibit its action by binding to LSD1. DUXAP10 directly inhibits the methylation of p21 and PTEN by LSD1, thereby promoting CRC cell proliferation 90. In pancreatic cancer, the lncRNA IRAIN inhibits the expression of the downstream target KLF2 by binding to LSD1 and EZH2^91^. Eventually, lncRNAs can also influence the activity of methylation enzymes by regulating their epigenetic modifications. Linc-GALH indirectly regulates the methylation status and expression of Gankyrin by promoting DNMT1 degradation by regulating the ubiquitination status of DNMT1 in HCC cells. Gankyrin is closely associated with the development and metastasis of HCC 92, 93. To summarize, lncRNAs play a role in gastrointestinal tumors by indirectly influencing the methylation of downstream targets by regulating methylation enzyme expression and activity. However, the way lncRNAs regulate methylation enzymes is complex and more research is required to understand their mechanisms of effect.

### 4.3 circRNA regulates methylation modification

CircRNAs are a class of circular ncRNAs that are more stable than linear RNAs and are rich in miRNA binding sites 94. CircRNAs, therefore, act mainly as miRNA sponges, and in addition, circRNAs can regulate the translation and post-translational modifications of many proteins 95, 96. CircRNAs have been found to regulate the expression of methylesterase through miRNA sponges in a variety of digestive tumors. For example, in colorectal cancer, Circ_0084615 upregulated DNMT3A expression through the sponge miR-599 to promote the proliferation, migration, and invasion of colorectal cancer cells 97. Similarly, Circ_0000467 inhibited miR-651-5p, and DNMT3B was a direct target of miR-651-5p. Knockdown of circ_0000467 upregulated DNMT3B expression and suppressed the malignant phenotype of colorectal cancer cells 98. In HCC, circ_0008583 also promoted HCC cancer progression by inhibiting miR-1301-3p and up-regulating METTL3 99. In addition to acting as miRNA sponges, circRNAs can also regulate digestive tumor progression in other ways. In EBV-associated gastric cancer, circPRMS1, produced by EBV, promotes the proliferation, migration, and invasion of gastric cancer cells. The mechanism is that circPRMS1 binds directly to Sam68 and activates the recruitment of Sam68 to the promoter region of METTL3 to promote its transcription 100. In addition, it was found that circPAR1 encapsulated by exosomes could directly bind to eIF3h and inhibit its interaction with METTL3. The oncogene BRD4's translation depends on the complex of METTL3 and eIF3h. circPAR1 indirectly downregulated the expression of BRD4 to inhibit the development of colorectal cancer 101. In conclusion, there are few studies on the regulation of methylation enzymes by circRNAs in gastrointestinal tumors, and circRNAs mainly regulate methylation enzymes through sponge miRNAs. Further mechanisms of CircRNA regulation of methylesterase remain to be explored.

## 5. Association of ncRNAs with methylesterases in various gastrointestinal tumors

In gastrointestinal tumors, a variety of ncRNAs modified by methylation and methylation enzymes regulated by ncRNAs are profoundly associated with tumor prognosis and clinical features. This section summarizes the relationship between ncRNAs and associated methylesterases and their clinical characteristics and prognosis in various GI tumors (Table 3). This suggests that ncRNAs and methylesterases may serve as key diagnostic markers or therapeutic targets in the following tumors.

### 5.1 Esophageal Cancer

The incidence and mortality rate of esophageal cancer remains high, with more than 600,000 people diagnosed worldwide each year 102. Increasingly, ncRNAs and methylation modifications have been found to play an important role in esophageal cancer. Zeng et al. found that low expression of miR-124-3p was highly correlated with the TNM stage and differentiation of ESCC. The methylation of miR-124-3p by DNMT1 played a key role in the regulation of miR-124-3p expression 43. This suggests that miR-124-3p and DNMT1 may be important prognostic markers for ESCC. In addition, upregulation of LINC00022 was significantly associated with poorer OS in ESCC patients, implying that LINC00022 is a poor prognostic factor. And LINC00022 was upregulated by m6A methylation of FTO 65. In 45 patients with esophageal cancer, lncRNA CASC15 was highly expressed in tumor tissues, and its expression was significantly associated with low OS, TNM stage, and lymphatic metastasis in patients. CASC15 was also upregulated by m6A methylation of FTO 103. In conclusion, ncRNAs modified by methylation are closely related to the poor prognosis of esophageal cancer. The selection of targeted drugs targeting methylating enzyme-associated ncRNAs may be effective in treating esophageal cancer.

### 5.2 Gastric cancer

Gastric cancer has the fifth highest incidence of all malignancies and has a poor prognosis, with a 5-year survival rate of less than 30% 104, 105. The development of gastric cancer is associated with many factors, including diet 106, H. pylori infection 107, and epigenetic changes 108. Because there are no apparent symptoms in the early stages of gastric cancer, patients are often diagnosed in the late stages of gastric cancer and therefore have a low 5-year survival rate 109. Currently, the main treatments for gastric cancer are surgical resection and chemotherapy, but the results are still poor 110. Therefore, there is a need to find an effective therapeutic target or early diagnostic marker. METTL14 was found to be upregulated in GC and significantly correlated with poor OS, TNM stage, and lymphatic metastasis in GC patients. The oncogenic effect of METTL14 was achieved by methylation of circORC5 111. In addition, high expression of METTL3 was found to be associated with lymphatic metastasis in gastric cancer patients, and METTL3 could act through the methylation of miR-17-92 56. In addition to methylesterases, many ncRNAs regulating methylation modifications have been linked to GC prognosis. LINC00240 was significantly upregulated in gastric cancer and was associated with poor patient survival, TNM stage, and distant metastasis 71. And LINC00240 could indirectly upregulate METTL3. These two studies suggest that the mutual regulation of ncRNAs and METTL3 has an important impact on the prognosis of gastric cancer patients. In addition, the upregulation of lncRNA DLEU1 was also associated with lymphatic metastasis and TNM stage of gastric cancer patients. As well as, DLEU1 could promote the methylation of LSD1 84.

### 5.3 Colorectal cancer

Colorectal cancer is the second most common cancer worldwide, and its incidence is expected to more than double by 2035 112, 113. The high incidence of colorectal cancer is mainly attributed to its asymptomatic early stage and the lack of practical screening tools 114. Current screening methods detect only 40% of cancer cases 113. Currently, the treatment options for colorectal cancer include surgery, chemotherapy, immunotherapy, and targeted therapy 115. The primary treatment for patients with advanced colorectal cancer is still chemotherapy, but the current resistance rate to chemotherapy is high 116. Therefore, there is an urgent need to identify markers and therapeutic targets that can diagnose colorectal cancer at an early stage. Many ncRNAs involved in methylation modifications were found to be closely related to the clinical prognosis of colorectal cancer. For example, LINC01605 is highly expressed in colorectal cancer, and its expression correlates with tumor stage, lymph node metastasis, and distant metastasis of colorectal cancer patients. High expression of LINC01605 predicts poor overall survival, and LINC01605 can promote the function of METTL3 to exert oncogenic effects 87. Furthermore, METTL3 downregulated LINC01559 expression, and low LINC01559 expression was also associated with TNM stage, lymph node metastasis, and distant metastasis in colorectal cancer patients 58. Moreover, HNF1A-AS1, circQSOX1, and circ1622 are all highly expressed in colorectal cancer and associated with poor prognosis in colorectal cancer patients, and they are all upregulated by METEL3 methylation 59, 60, 117. High expression of METTL3 has been shown to be highly expressed in colorectal cancer and correlated with low OS in patients. These results confirm that METTL3 and its mutually regulated ncRNA molecules are strongly associated with the prognosis of colorectal cancer patients and have promise as diagnostic markers and therapeutic targets for CRC.

### 5.4 Liver cancer

Liver cancer is the third most common cause of cancer deaths. In 2020 a total of 83,000 people died from liver cancer worldwide 118. Ninety percent of all primary liver cancers are hepatocellular carcinoma (HCC) 119. Risk factors for HCC include hepatitis B virus, hepatitis C virus, and alcohol consumption 120. It was found that ncRNAs and their associated methylation enzymes are aberrantly expressed in hepatocellular carcinoma and correlated with patient prognosis. MiR-378a-3p was downregulated by DNMT1 methylation in HCC. Low expression of MiR-378a-3p was associated with poor OS, tumor thrombosis, and (microvascular density) MVD in patients 46. As a highly vascular tumor, high MVD in hepatocellular carcinoma represents enhanced angiogenesis, and its prognostic significance in HCC is significant 121, 122. Alternatively, LncRNAs SNHG22, DDX11-AS1, and Linc-GALH were found to be upregulated in HCC and associated with poor prognosis and clinical features of hepatocellular carcinoma. All three lncRNAs can act by regulating DNMT1 methylation oncogenic factor 45, 89, 92. LncRNA BZRAP1-AS1 is highly expressed in HCC and strongly correlates with tumor size, microvascular invasion, and TNM stage. DNMT3B is upregulated by BZRAP1-AS1 88. Besides, miR-29c-3p can inhibit DNMT3B, and low expression of miR-29c-3p is associated with intrahepatic metastasis and tumor multiplicity in HCC patients 75. Many ncRNAs regulating methylesterase are associated with poor prognosis of HCC, and the discovery of more ncRNAs in relation to methylesterase regulation may help to find appropriate diagnostic markers for HCC.

### 5.5 Pancreatic cancer

Pancreatic cancer has the worst prognosis of all cancers, although its incidence is slightly lower than that of other digestive system tumors 123. Pancreatic cancer's mortality to incidence ratio is as high as 94% 124. The mortality rate of most other tumors has been decreasing year by year, but the mortality rate of pancreatic cancer has remained almost unchanged 125. There is still a lack of effective treatment for pancreatic cancer 126. The LncRNA IRAIN was found to be highly expressed in pancreatic cancer, and its high expression predicted advanced pathological stage, larger tumor size, and lymph node metastasis. target genes by binding to LSD1 and EZH2 91. Targeting IRAIN, LSD1, or EZH2 may be a potential approach to treat pancreatic cancer. There are few studies on the interaction between ncRNAs and methylation modifications in PC. More studies are needed to explore the potential value of ncRNAs and methylation modifications in the clinical prognosis of pancreatic cancer.

## 6. Potential clinical value of ncRNAs involved in methylation modifications

**Figure 3:**
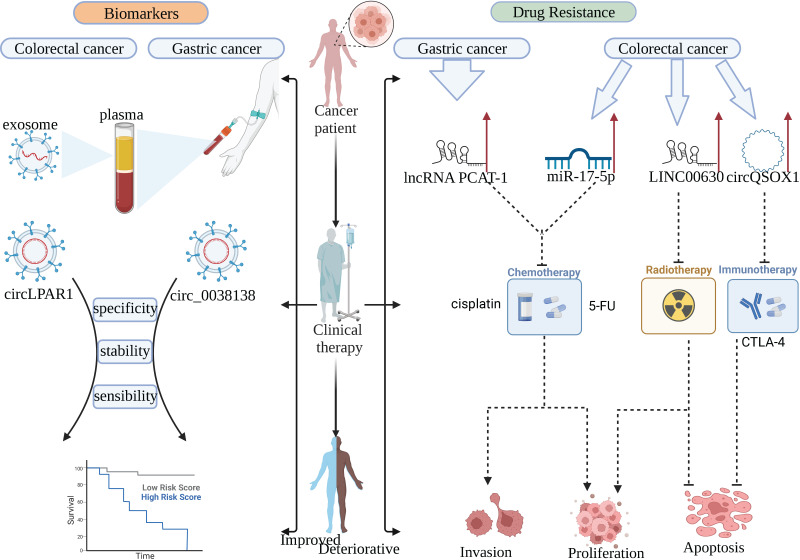
NcRNAs can be used as biomarkers for patients and targets for drug resistance therapy in clinical practice. circRNA in plasma exosomes has high stability, sensitivity, and specificity, which can replace the existing tumor markers. lncRNA PCAT-1, miR-17-5p, LINC00630, and circQSOX1 play important drug resistance roles in anti-chemotherapy, anti-radiotherapy, and anti-immunotherapy, respectively.

With the continuous research on the clinical application of ncRNA, we found that ncRNAs have a considerable prospect in gastrointestinal cancer, which is expected to be used as clinical tumor biomarkers, therapeutic targets, and targeted targets for anti-drug resistance (Figure 3).

### 6.1 Serve as biomarkers

The discovery of more effective diagnostic markers can help diagnose patients with gastrointestinal tumors early so that patients can receive early treatment 127. At the same time, effective prognostic markers can reflect the malignancy of patients' tumors and determine the recurrence and metastasis of tumors 128. With the in-depth study of ncRNAs and related methylation enzymes, both of them are expected to be effective diagnostic and prognostic markers in gastrointestinal tumors. In colorectal cancer, circLPAR1, which is encapsulated by exosomes, was found to have reduced expression in the plasma of patients. Its low expression was associated with poor OS in colorectal cancer patients. More importantly, it was found that exosomal circLPAR1 was stable in plasma, and its expression did not change significantly after repeated freeze-thawing or long-term storage at room temperature. CircLPAR1 was used to differentiate colorectal patients from normal controls with better efficiency than CEA and CA19-9 101. CEA and CA19-9 are currently and popularly used diagnostic markers for colorectal cancer 129. This suggests that circLPAR1 holds promise as an effective diagnostic and prognostic marker for colorectal cancer. The mechanism is that circLPAR1 exerts its oncogenic effect by inhibiting METTL3. And METTL3 was also found to be highly expressed in the plasma of CRC patients 130. Similarly, increased expression of circ_0038138 was found in plasma exosomes of gastric cancer patients. Circ_0038138 indirectly downregulated EZH2 through miR-198 131. In another study, EZH2 was found to be present in small extracellular vesicles (sEV) of gastric cancer cells, and its expression was significantly higher in the plasma of patients than in healthy subjects 132. On the other hand, METTL3 can also be used as a marker to detect drug sensitivity to guide drug administration. It was found that gastric cancer patients with high METTL3 were more sensitive to everolimus. This may be since everolimus, as an mTOR inhibitor, blocks the METTL3/miR-17-92/PTEN/AKT/mTOR axis. And high expression of METTL3 suggested activation of the AKT/mTOR pathway 56. Marker assays against plasma are noninvasive and easily promoted screening tool, and assays against specific ncRNAs and methylesterases in serum are expected to be an effective screening tool 133. It is believed that with more in-depth studies on ncRNAs and related methylesterases, more effective diagnostic and prognostic markers will be found.

### 6.2 Serve as therapeutic targets

Gastrointestinal tumors are solid tumors, and the current treatment is still based on surgery, chemotherapy, and radiotherapy 134-136. In recent years, with the continuous exploration of molecular biology, targeted therapies targeting specific genes and cells have been developed. Among them, the delivery of siRNA to tumor cells through nanomaterials has become a promising approach for cancer therapy 137. Du et al. demonstrated that delivery of circMDK siRNA via poly (β-amino esters) (PAEs) to a liver cancer model could effectively inhibit tumor progression. CircMDK is upregulated in hepatocellular carcinoma by m6A methylation modifications 11. However, the mechanism of how circMDK is modified by methylation is unclear. Further investigation of the mechanism of action of circMDK on m6A methylation-modified enzymes by demethylating circMDK may be a new approach to target circMDK for the treatment of HCC.

### 6.3 Serve as a target for improving drug resistance

Drug resistance has been a significant challenge in the treatment of tumors, and eliminating tumor resistance through a combination of drugs is a promising strategy for the treatment of tumors 138. It was found that the knockdown of lncRNA PCAT-1 could re-sensitize drug-resistant GC cells to cisplatin. And PACT-1 is resistant to GC cells by inhibiting PTEN through binding to EZH2. Targeting PACT-1 is a promising therapeutic approach for patients with cisplatin-resistant gastric cancer 139. Similarly, in colorectal cancer, overexpression of miR-17-5p regulated by METTL14 methylation leads to 5-FU resistance in CRC cells. Knockdown of miR-17-5p could improve the sensitivity of CRC cells to 5-FU 140. In addition to chemoresistance, radioresistance is also an important factor affecting patient outcomes 141. High expression of LINC00630 was found to be associated with radioresistance in CRC. This was mainly achieved by the inhibition of BEX1 by LINC00630 through EZH2. Targeted inhibition of LINC00630 significantly improved the radioresistance of CRC cells 142. Immunotherapy is also an emerging therapeutic strategy in recent years, as CTLA-4 is an immune checkpoint that is expressed mainly on Treg cells and mediates their immunosuppressive capacity 143. Anti-CTLA-4 therapy is highly effective in cancer patients who are sensitive to it, but unfortunately, most patients do not respond to this therapy. Therefore, addressing CTLA-4 resistance in tumor cells is an urgent issue at present 144. It was found that circQSOX1 upregulation in colorectal cancer inhibited the anti-CTLA-4 treatment response in colorectal cancer cells. Knockdown of circQSOX1 could effectively improve the sensitivity of colorectal cancer tumors to CTLA-4. Mechanistically, METTL3 upregulates circQSOX1 via m6A methylation, and circQSOX1 indirectly promotes PGAM1 expression through sponge miR-326 and miR-330-5p to make colorectal cancer cells exhibit resistance to immunotherapy 59. Further exploration of the regulatory mechanisms between ncRNAs and methylation modifications is expected to address the drug resistance problem in clinical treatment and help identify more effective therapeutic targets.

## 7. Conclusion

As methylation modifications of DNA, histones, and RNA have been intensively studied, there is increasing evidence for the important role of methylation modifications in cancer regulation. With the rapid development of transcriptomics, the role of ncRNAs in gastrointestinal tumors has been gradually revealed. As two essential regulators of epigenetic modifications, the interplay between ncRNAs and methylation modifications has been shown to play an important role in regulating the malignant phenotype of GI tumors, influencing the prognosis of tumor patients and drug resistance. DNA and histone methylation modifications can directly regulate the transcription of ncRNAs, and RNA methylation can regulate the degradation, synthesis, and processing of ncRNAs. In addition, ncRNAs can also directly or indirectly regulate the activity of methylation enzymes and further regulate the expression level of cancer-related genes in various ways. At the same time, ncRNAs and methylation modifications can also form a feedback loop to promote tumor cell proliferation and metastasis. For example, the oncogenic effect of CircGPR137B in HCC is achieved by upregulating FTO to develop a positive feedback loop. In clinical applications, some ncRNAs that can be stably expressed in plasma have shown potential value as effective diagnostic markers. In addition, siRNAs and methylation enzyme inhibitors targeting ncRNAs have shown great promise in tumor therapy. It is believed that further exploration of the regulatory mechanisms of ncRNAs and methylation modification will lead to the discovery of more effective diagnostic markers and therapeutic targets for eventual clinical application.

## Figures and Tables

**Table 1:** The molecular mechanisms and biological processes involved in the regulation of ncRNAs by methylases

Type	methylase	ncRNA	Trend of regulation	Axis/pathway	Biological activities	Reference
DNA methylation	DNMT3B	miR-149	down-regulated	DNMT3B/miR-149/RNF2/Wnt/β-catenin	promote migration, invasiveness and EMT	42
	DNMT1	miR-124-3p	down-regulated	DNMT1/miR-124-3p/BCAT1	promote proliferation and migration	43
	DNMT1	miR-152-3p	down-regulated	DNMT1/miR-125-3p/TMSB10	promote proliferation, migration, invasion and inhibit apoptosis	47
	DNMT1	miR-378a-3p	down-regulated	DNMT1/miR-378a-3p/TRAF1/NF-κB positive feedback loop	promote angiogenesis	46
	DNMT1	miR-16-5p	down-regulated	SNHG22/EZH2/DNMT1/miR-16-5p	promote proliferation, migration, invasion, and angiogenesis	45
	DNMT1	miR-34a	down-regulated	DNMT1/miR-34a/Notch		41
Histone methylation	EZH2	miR-200c	up-regulated	EZH2/miR-200c	promote migration, EMT	49
	LSD1	miR-142-5p	up-regulated	LSD1/miR-142-5p/CD9	promote migration, EMT	53
	R1ZI	HOTAIRM1	up-regulated	R1ZI/HOTAIRM1	promote proliferation, migration and Invasion	30
	EED	miR-338-5p	down-regulated	EED/miR-3385p/METTL3/CDCP1	promote proliferation and invasion	51
RNA methylation	FTO	LINC00022	up-regulated	FTO/LINC00022/p21	promote cell-cycle and proliferation	65
	FTO	CASC15	up-regulated	FTO/CASC15/SIM2	promote proliferation and inhibit apoptosis	103
	FTO	circGPR137B	up-regulated	circGPR137B/miR-4739/FTO feedback loop	promote growth and invasion	66
	WTAP	circCMTM3	up-regulated	WTAP/circCMTM3/IGF2BP1/PARK7	inhibit ferroptosis	64
	METTL14	circORC5	down-regulated	METTL14/circORC5/miR-30c-2-3p/AKT1S1	inhibit proliferation, invasion	111
	METTL14	LINC01320	up-regulated	METTL14/LINC01320/miR-495-5p/RAB19	promote proliferation, migration, and invasion	67
	METTL14	XIST	down-regulated	METTL14/YTHDF2/XIST	inhibit proliferation and invasion	62
	METTL14	miR-17-5p	down-regulated	METTL14/miR-17-5p/MFN2	promote apoptosis	140
	METTL14	circFUT8	up-regulated	miR-628-5p/METTL14/circFUT8/miR-552-3p/CHMP4B	promote proliferation and inhibit apoptosis	63
	METTL3	circ1662	up-regulated	METTL3/circ1662/YAP1	promote invasion, migration and EMT	60
	METTL3	THAP7-AS1	up-regulated	SP1/METTL3/THAP7-AS1/CLUB4/ miR-22-3p,miR-320a/ PI3K/AKT	promote proliferation, migration and invasion	57
	METTL3	miR-17-92	up-regulated	METTL3/miR-17-92/PTEN, TMEM127/AKT/mTOR	promote proliferation, migration and invasion	56
	METTL3	LINC01559	down-regulated	METTL3/LINC01559/miR-106b-5p/PTEN	promote proliferation and metastasis	58
	METTL3	HNF1A-AS1	up-regulated	METTL3/HNF1A-AS1/IGF 2BP2/CCND1	promote migration, invasion, cell cycle and angiogenesis	117
	METTL3	circ QSOX1	up-regulated	METTL3/circ QSOX1/miR-326 and miR-330-5p/PGAM1	promote proliferation, migration and invasion	59
	METTL3	circ_0058493	up-regulated	METTL3/circ_0058493/YTHDC1	promote proliferation and metastasis	61

**Table 2:** The molecular mechanisms and biological processes involved in the regulation of methylases by ncRNAs

Type	ncRNA	Methylase	Trend of regulation	Axis/pathway	Biological activities	Reference
miRNA	miR-492	DNMT3B	repress	miR-492/DNMT3B	promote proliferation, metastasis and stemness	69
	miR-338-5p	METTL3	repress	LINC00240/miR-338-5p/METTL3	inhibit proliferation, migration, invasion and promote apoptosis	71
	miR-4429	METTL3	repress	miR-4429/METTL3/SEC62	inhibit proliferation and promote apoptosis	70
	miR-515-5p	DNMT1	repress	circ_0040809/miR-515-5p/DNMT1	inhibit proliferation, migration and promote apoptosis	72
	miR-34a-5p	DNMT3A	repress	miR-34a-5p/cMYC/DNMT3a/PTEN	inhibit proliferation, migration, invasion and promote apoptosis	73
	miR-96	FTO	repress	miR-96/AMPKα2/FTO/MYC	promote proliferation, migration, invasion and inhibit apoptosis	74
	miR-29c-3p	DNMT3B	repress	miR-29c-3p/DNMT3B/LATS1	inhibit proliferation, migration, invasion and promote apoptosis	75
	miR-144-3p	WTAP	repress	LINC00839/miR-144-3p/WTAP	promote proliferation, invasion and migration	77
	miR‑29b	DNMT3B	repress	miR‑29b/DNMT3B	inhibit proliferation and promote apoptosis	78
LncRNA	LINC00858	FTO	promote	LINC00858/ZNF184/FTO/MYC	promote proliferation, apoptosis, cell cycle, migration and invasion	81
	LINC00467	DNMT1	promote	LINC00467/DNMT1/Reprimo	proliferation, apoptosis, migration and invasion	83
	SAMD12-AS1	DNMT1	promote	SAMD12-AS1/DNMT1/p53	promote proliferation and cell cycle	82
	DLEU1	LSD1	promote	DLEU1/LSD1/KLF2	promote proliferation and inhibit apoptosis	84
	LINC00337	DNMT1	promote	LINC00337/DNMT1/CNN1	promote tumorigenesis and angiogenesis	85
	LINC01605	METTL3	promote	SMYD2-EP300/LINC01605/METTL3/SPTBN2	promote proliferation and metastasis	87
	DDX11-AS1	DNMT1, EZH2	promote	DDX11-AS1/DNMT1, EZH2/LATS2	promote proliferation, migration, and invasion	89
	Linc-GALH	DNMT1	repress	Linc-GALH/DNMT1/Gankyrin	promote migration and invasion	92
	HCP5	DNMT3A	repress	HCP5/miR-29b-3p/DNMT3A/AKT	promote proliferation, migration, and invasion	80
	BZRAP1-AS1	DNMT3B	promote	BZRAP1-AS1/DNMT3B/THBS1	promote proliferation, migration and angiogenesis	88
	DUXAP10	LSD1	promote	DUXAP10/LSD1/p21, PTEN	promote proliferation, apoptosis and cell cycle	90
	IRAIN	LSD1, EZH2	promote	IRAIN/LSD1, EZH2/KLF2,P15	promote cell cycle and inhibit apoptosis	91
CircRNA	circRPMS1	METTL3	promote	circRPMS1/Sam68/METTL3	promote proliferation, migration, invasion and inhibit apoptosis	100
	Circ_0084615	DNMT3A	promote	Circ_0084615/miR-599/DNMT3A	promote proliferation, migration and invasion	97
	Circ_0000467	DNMT3B	promote	Circ_0000467/miR-651-5p/DNMT3B	promote growth, migration and invasion	98
	circLPAR1	METTL3	promote	circLPAR1/eIF3h/METTL3/BRD4	promote proliferation, migration and invasion	101
	circ_0008583	MTTTL3	promote	circ_0008583/miR-1301-3p/METTL3	promote proliferation, migration and invasion	99

**Table 3:** Expression and clinical features of ncRNAs or methylases in patients with GI cancer

Cancer type	ncRNAs/methylase	Expression	Clinical features	Reference
ESCC	miR-124-3p	down	TNM stage and differentiation grade	43
ESCC	LINC00022	up	OS	65
ESCC	CASC15	up	OS, TNM stage and lymphatic metastasis	103
GC	METTL14	down	OS	111
GC	LINC01320	up	OS	67
GC	THAP7-AS1	up	OS	57
GC	METTL3	up	lymph node metastasis	56
GC	LINC00240	up	OS, TNM stage,distant metastasis and lymph nodes metastasis	71
GC	SAMD12-AS1	up	OS	82
GC	DLEU1	up	lymph node metastasis, TNM stage	84
CRC	METTL14	up	RFS, larger tumor size, lymphatic invasion, remote metastasis and TNM stage	62
CRC	circ1662	up	OS, lymph node metastasis and vascular invasion	60
CRC	LINC01559	down	TNM stage, lymphatic metastasis and distant metastasis	58
CRC	HNF1A-AS1	up	OS, higherpathological stage (III/IV), lymph node metastasis and distant metastasis	117
CRC	circQSOX1	up	OS	59
CRC	circ_0040809	up	OS	72
CRC	LINC01605	up	OS, lymph node metastases, distal metastases and advanced tumor stage	87
CRC	DUXAP10	up	tumor sizes, TNM stages and lymph node metastasis	90
CRC	Circ_0084615	up	OS	97
CRC	circLPAR1	down	OS	101
HCC	miR-378 a-3p	down	OS, thrombosis of tumor blood vessel and MVD	46
HCC	SNHG22	up	OS	45
HCC	circGPR137B	down	OS	66
HCC	CircCMTM3	up	OS	64
HCC	hsa_circ_0058493	up	OS	61
HCC	miR-29c-3p	down	OS, tumor size, multiplicity, and intrahepatic metastasis	75
HCC	LINC00839	up	OS, tumor size, lymph node metastasis and poor tumor differentiation	77
HCC	DDX11-AS1	up	serum AFP levels and tumor stage	89
HCC	Linc-GALH	up	intrahepatic metastasis, vascular invasion and distant metastasis	92
HCC	HCP5	up	large tumors, metastasis, high histological grade tissues, and recurrence	80
HCC	BZRAP1-AS1	up	tumor size, microvascular invasion and TNM stage	88
PC	IRAIN	up	advanced pathological stage, larger tumor size, and lymph node metastasis	91
